# Characteristics of Human Endometrial Stem Cells in Tissue and Isolated Cultured Cells: An Immunohistochemical Aspect

**DOI:** 10.7508/ibj.2016.02.006

**Published:** 2016-04

**Authors:** Mehri Fayazi, Mojdeh Salehnia, Saeideh Ziaei

**Affiliations:** 1 Dept. of Anatomy, Tarbiat Modares University, Tehran, Iran;; 2 Dept. of Midwifery, Tarbiat Modares University, Tehran, Iran

**Keywords:** Endometrium, Immunohistochemistry, Mesenchymal stem cells

## Abstract

**Background:**

The aim of this study was to investigate the percentage of the stem cells population in human endometrial tissue sections and cultured cells at fourth passage.

**Methods:**

Human endometrial specimens were divided into two parts, one part for morphological studies and the other part for in vitro culture. Full thickness of human normal endometrial sections and cultured endometrial cells at fourth passage were analyzed via immunohistochemistry for CD146 and some stemness markers such as Oct4, Nanog, Sox2, and Klf4 and the expression of typical mesenchymal stem cell markers CD90, CD105.

**Results:**

11.88±1.29% of human endometrial cells within tissue sections expressed CD146 marker vs. 28±2.3% of cultured cells, CD90 and CD105 were expressed by functionalis stroma (85±2.4 and 89±3.2%) than basalis stroma (16±1.4 and 17±1.9%), respectively (*P*<0.05). Oct4 and Nanog-expressing cells comprise 1.43±0.08 and 0.54±0.01% of endometrial stromal cells in endometrial sections vs. 12±3.1% and 8±2.9% of cultured cells, respectively. They reside near the glands in the basal layer of endometrium. Sox2 and Klf4 were not commonly expressed in tissue samples and cultured cells. CD9 and EpCAM were expressed by epithelial cells of the endometrium, rather than by stroma or perivascular cells.

**Conclusion:**

The human endometrial stem cells and pluripotency markers may be localized more in basalis layer of endometrium. The immunostaining observations of endometrial cells at fourth passage were correlated with the immunohistochemistry data.

## INTRODUCTION

Adult stem cells are rare multipotent cells that have been identified in several adult tissues such as intestine^[^^1^^]^, skin^[^^2^^]^, muscle^[^^3^^]^, blood^[^^4^^]^, nervous system^[^^5^^]^, and endometrium^[^^6^^]^. Human endometrial stem cells were recognized for the first time by Chan *et al.*^[^^7^^]^ in 2004. Some of their properties are self-renewal, high proliferative potential, ability to differentiate into one or more lineages, clonogenicity, and tissue reconstitution *in vivo*^[^^8^^, ^^9^^]^. It is extremely difficult to identify these cells in tissues because they do not have certain morphological features and specific markers^[^^10^^,^^11^^]^. 

 During every menstrual cycle of a woman, the endometrium physiologically undergoes cyclical changes such as self-renewal, proliferation, differentiation, and shedding off ^[^^7^^,^^12^^]^. Endometrial regeneration also occurs after each endometrial incision and pregnancy^[^^13^^,^^14^^]^. These characteristics of the endometrium have suggested the presence of a low number of endometrial-derived stem cell (EnSC) populations that seem to be responsible for its remarkable regeneration ability. 

EnSCs are isolated easily, expand rapidly as well as produce a higher clonogenicity and a non-invasive source that make it a great therapeutic potential as autologous stem cell alternative in women^[^^15^^]^. Phenotypically, EnSCs appear to share some markers with mesenchymal stem cells (MSCs) such as CD90 and CD105^[^^16^^]^. A recent study found a novel single marker, CD146, that was able to isolate stem cells in human endometrium^[^^17^^]^. However, the localization and percentage of some stemness markers in human endometrial tissue sections and cultured endometrial cells remain unclear. Therefore, further detailed studies are necessary for their identification. The aim of this study was to investigate the presence and the percentage of stem cells population by immune-histochemistry in human endometrial tissue sections and immunocytochemistry in human endometrial cultured cells at fourth passage. 

## MATERIALS AND METHODS

**Human endometrial tissues**

Human endometrial specimens were obtained from five healthy women (aged between 30-45 years) after hysteroscopy for non-endometrial benign pathological condition. These women had not taken exogenous hormones for three months prior to surgery. The use of the human specimens was approved by the Ethics Committee of Medical Faculty of Tarbiat Modares University, Tehran, Iran. The normality of the endometrial tissue was proved by histological examination according to well-established histological criteria of normal menstrual cycle and confirmed by an experienced histopathologist. The proliferative stage was selected for all of the specimens in order to synchronize them. 

**Experimental design**

Each endometrial specimen was divided into two parts, one part for morphological and immune-histochemical studies and the other part for isolation and cultivation of endometrial cells, followed by immunocytochemical study.

**Morphological assessments of endometrial samples**

 Full thickness of human normal endometrial tissues (n=5) were fixed in 10% formalin, processed and embedded in paraffin wax and then sectioned at 5 µm^[^^18^^]^. After routine hematoxylin and eosin staining, the morphology of endometrial sections was observed under a light microscope. Other sets of paraffin sections of endometrial tissue were collected and considered for immunohistochemistry. 

**Immunohistochemistry of human endometrial sections**

The paraffin sections were put on gelatin-coated slides (n=3 per each sample). After deparaffinization and rehydration with decreasing gradient concentration of ethanol, the sections were washed in phosphate buffer saline (PBS) for 5 min. Endogenous peroxidase activity was blocked with a 0.3% H_2_O_2_ solution in methanol for 20 min. Subsequently, antigen retrieval was carried out in a 0.01-M sodium citrate solution (pH 6) in an oven at 95°C for 20 min. The slides were cooled down to room temperature for 20 min and washed with deionized water and PBS, respectively. Then they were blocked by blocking solution (Bioidea, Iran) for 10 min. The sections were incubated separately with a panel of primary antibodies, including CD146, CD90, CD105, Oct4, Nanog, Sox2, Klf4, CD9, and EpCAM at 37°C for 30 min and washed three times in PBS. Secondary antibodies (Bioidea, Iran) were used for 10 min and washed again with PBS. Next, the sections were treated with horseradish peroxidase (Bioidea, Iran) for 10 min and diaminobenzidine solution (Bioidea, Iran), respectively^[^^19^^]^. Finally, they were counterstained by hematoxylin, dehydrated and mounted. Immuno-staining for each antibody was conducted on three tissue samples, and three sections per each sample were immunostained. Then microscopic fields were observed for evaluation of the stained cells. Brown colors showed the location of antigen in these cells. 

**Isolation of human endometrial cells**

Preparation of isolated human endometrial cells was carried out according to the method described earlier by Chan *et al.*^[^^7^^]^. Briefly, human endometrial tissue was scraped from the myometrium and washed in PBS. Then the tissue was minced in a medium containing Dulbecco modified eagle medium/Ham's F-12 (DMEM/F-12; Invitrogen, UK) supplemented with 100 mg/ml penicillin G sodium and 100 mg/ml streptomycin sulfate B (Invitrogen, UK). Next, tissues fragments were digested and dissociated into a single cell using collagenase type 3 (300 µg/ml; Sigma, Germany), deoxyribonuclease type I (40 µg/ml; Sigma, Germany), and mechanical methods at 37°C for 60-90 min. Next, to remove glandular and epithelial com-ponents, they were pipetted and passed sequentially through meshes of 150, 100, 40 sieve (BD Biosciences, Erembodegem, Belgium), respectively^[^^20^^]^.

***In vitro*** **culture of human endometrial cells**

Purified endometrial stromal cells were seeded at 2 × 10^4^ cells per cm^2^ using DMEM/F-12 supplemented with 100 mg/ml penicillin G sodium and 100 mg/ml streptomycin sulfate B (Invitrogen, UK) and 5% fetal bovine serum (Invitrogen, UK) and then incubated in 5% CO_2_ at 37°C. The medium was changed every 2-3 days. The cells were passaged when cultures reached 80-100% confluency. For passaging, after washing with PBS, the cells were treated with 0.05% trypsin and 0.02% *ethylenediaminetetraacetic aci*d at 37°C for 3 min, and then complete DMEM was added to stop the enzyme reactions. Afterward, the cells were separated by centrifugation and then cultured at 37ºC for 24 h. After passage four, the trypsinized cells were cultured on a gelatin-coated glass coverslip at 37°C for 24 h, and then immunocytochemistry were performed.

**Immunocytochemical staining of human endometrial stromal cells**

The immunocytochemistry were carried out for CD146, CD90, CD105, Oct4, Nanog, Sox2, Klf4, CD9, and EpCAM markers after passage four. The medium was removed, and the cells were washed three times in PBS. After fixation with 4% paraformaldehyde at 4°C for 45 min, the cells were permeabilized with 0.3% Triton X-100 (Sigma, Belgium) for 15 min. For blocking unspecific binding site of antigens, the cells were rinsed with 10% bovine serum albumin in PBS for 60 min, and incubated at 4°C overnight with the primary antibodies that mentioned previously. The cells were then washed three times in PBS and incubated with secondary antibody (conjugated with FITC) for two hours, followed by washing in PBS^[^^21^^]^. Finally, for counterstaining, the cells were stained by 4',6-diamidino-2-phenylindole (Sigma, Germany) for 20 seconds to demonstrate the nuclei and observed under a fluorescent microscope (Olympus, Japan). The primary antibodies were deleted in negative controls.

**Statistical analysis**

The photograph of each section or coverslip was prepared and imported into Image J software (National Institutes of Health, Bethesda, USA). The rate of immunostained cells per nucleated cell was evaluated. Statistical analysis was conducted using SPSS17 software. The quantitative results in the present study were presented as means±standard errors of the means (SEM).

## RESULTS

**Histological examination and** **immunohisto-chemistry of human endometrium**

**Fig. 1:**
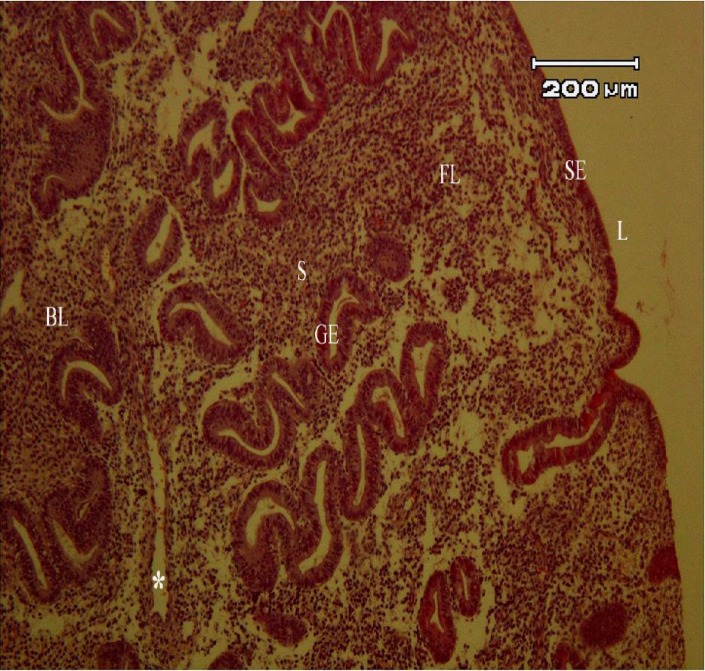
Light microscopic morphology of human endometrium using H & E staining. S, stroma; SE, surface epithelium; GE, glandular epithelium; BL, basalis layer; FL, functionalis layer; L, lumen; *, blood vessels

**Fig. 2:**
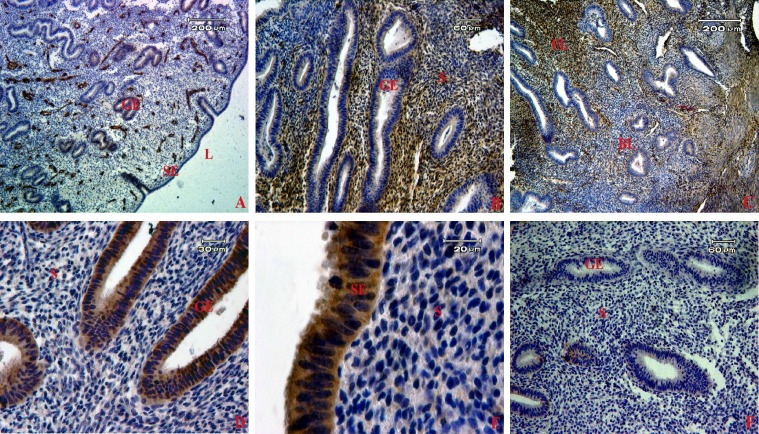
Localization of stem cell markers in human endometrial tissue samples. The brown color shows the expression of each marker by immunohistochemistry using diaminobenzidine as the substrate. A group of CD146 (A), CD90- (B) and CD105- (C) positive cells were localized in both basalis and functionalis stroma. The expression of CD9 (D) and EpCAM (E) were detected in epithelial cells. (F) Sections of endometrium were stained with secondary antibody- (without primary antibody) serving as a negative control in our study. S, stroma; SE, surface epithelium; GE, glandular epithelium; BL, basalis layer; FL, functionalis layer; L, lumen

**Fig. 3.**
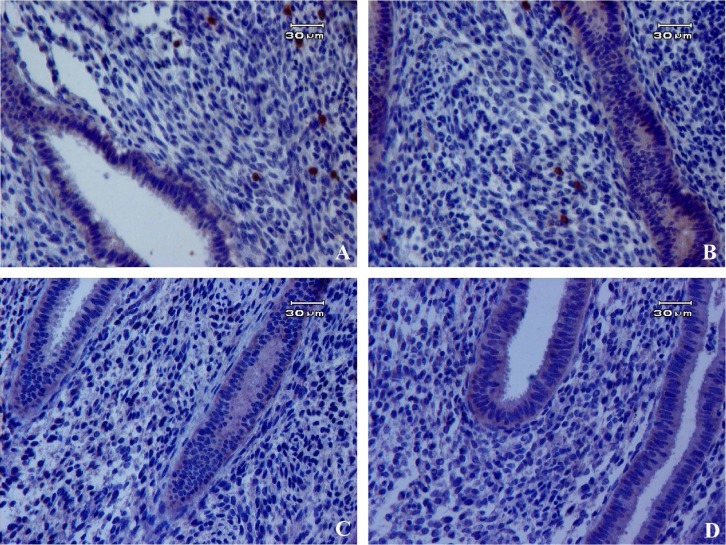
Immunohistochemistry of pluripotency markers in human endometrial tissues. Positive expression of Oct4 (A) and Nanog (B) was detected in the nucleus of endometrial cells but no expression for (C and D) Sox2 and Klf4 markers

The normal morphology of endometrial tissue at proliferative stage with hematoxylin and eosin indicated two remarkable layers of endometrium: basalis and functionalis (Fig. 1). The localization of CD146, CD90, CD105, Oct4, Nanog, Sox2, Klf4, CD9, and EpCAM markers in human endometrial tissues is shown in Figures 2 and 3. The intensity of immunoreactive cells at different parts of the endometrial tissue sections is summarized in Table 1.

Human endometrial CD146-positive cells were localized mainly in the stroma around the glands and perivascular location. The cells were more prominent in basalis than functionalis layers. No CD146-positive cells were observed within the surface and glandular epithelium (Fig. 2A). The proportion of CD146-positive cells was 11.88±1.29% in human endometrial tissues.

The immunohistochemistry showed a high protein expression of CD90 and CD105 markers specifically in stromal cells of human endometrium, but they were not expressed in the epithelial cells (Fig. 2B and 2C). The proportion of CD90- and CD105-positive cells in the functionalis layer was 85±2.4 and 89±3.2% and in basalis layer was 16±1.4 and 17±1.9%, respectively. 

These markers strongly were expressed in functionalis rather than basalis layer (*P*<0.05).

The most frequent location for Oct4- and Nanog-positive cells was in basalis stroma (Fig. 3A and 3B). Oct4 and Nanog staining was visible in the nucleus of the cells. Oct4 and Nanog-expressing cells were 1.43±0.08 and 0.54±0.01%, respectively in human endometrial tissues. Sox2 and Klf4-expressing cells were not detected in human endometrium (Fig. 3C and 3D). 

The results showed that all surface epithelial cells and glands were positive for epithelial markers, including CD9 and EpCAM markers in the sections (Fig. 2D and 2E). Moreover, some endometrial sections without primary antibody were considered as a negative control in our experiments to confirm the results (Fig. 2F). 

**The morphology of human cultured endometrial cells**

**Fig. 4:**
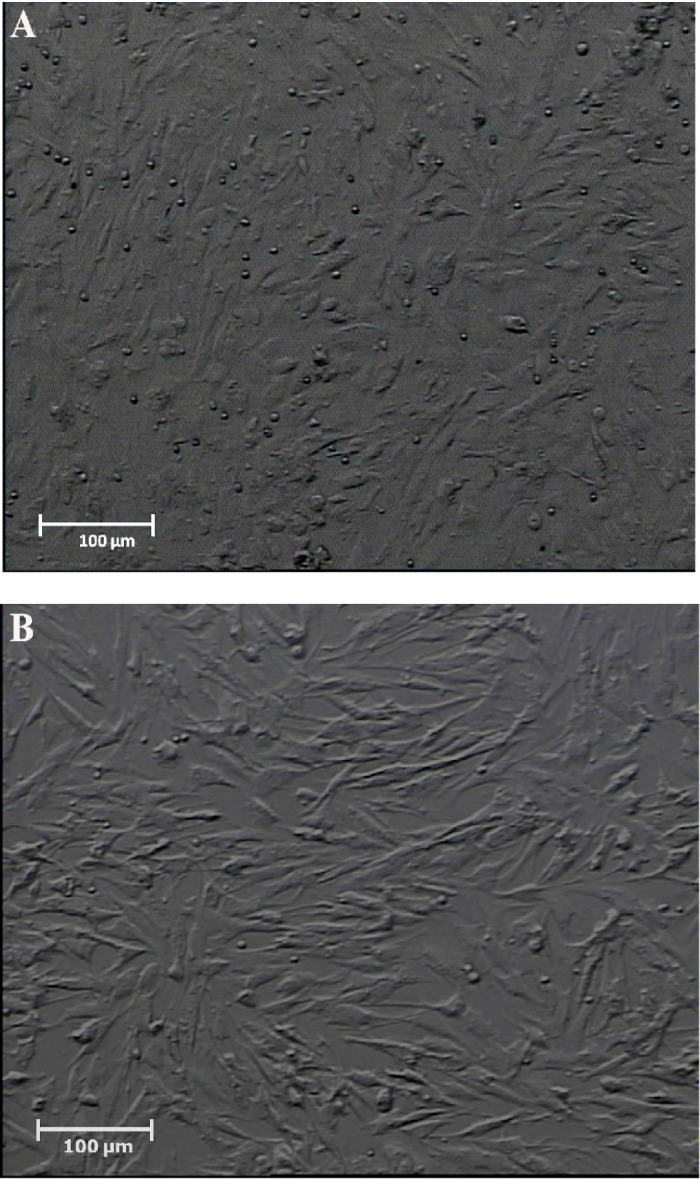
Phase-contrast photomicrograph of cultured human endometrial cells at passage 1 (A) and passage 4 (B). Cells usually appeared elongated and spindle-shape with round nuclei

The figures of cultured cells at fourth passage showed uniformed cells (Fig. 4). Cells usually appear elongated and spindle-shape with round nuclei. 

**Immuocytochemistry of cultured endometrial stromal cells**

**Fig. 5:**
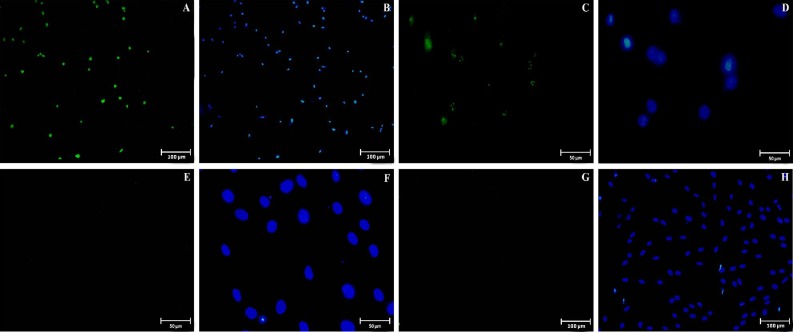
Immunostaining of cultured endometrial cells at fourth passage. The cells were immunostained for the primary antibodies against CD146 (A and B) as EnSCs, CD90 (C and D) and CD105 (E and F) markers that are mesenchymal stem cells. CD9 (G and H) and EpCAM (I and J) were used as epithelial markers. They were labeled with FITC-conjugated secondary antibody (green color shows positive reactions). The nuclei were immunostained (second and fourth column) with 6-diamidino-2-phenylindole (blue colors). (K and L) Negative control is also shown.

**Fig. 6:**
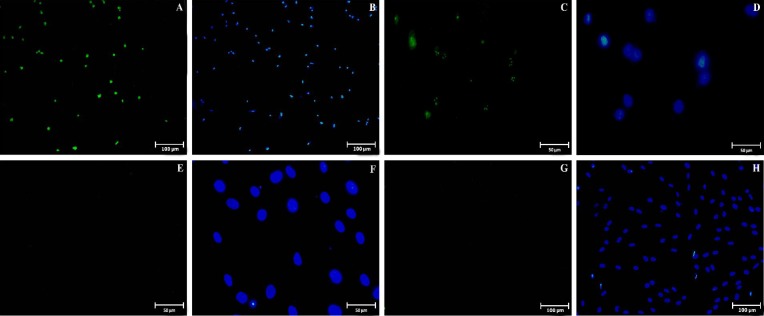
Immunostaining of pluripotency markers in cultured endometrial cells at fourth passage. Positive staining for Oct4 (A and B) and Nanog (C and D) markers were detected in endometrial cells. No staining were seen for Sox2 (E and F) and Klf4 (G and H) markers. The second and fourth column of the fluorescent photomicrographs represents the nuclei (blue colors: 6-diamidino-2-phenylindole) images from the same field of the immunofluorescence images

The proportion of CD146, CD90, CD105, Oct4, Nanog, Sox2, Klf4, CD9, and EpCAM markers in endometrial cells at fourth passage is summarized in Table 2. CD146 marker was expressed specifically by a population (28±2.3%) of endometrial stromal cells (Fig. 5A and 5B). The cells were found to be strongly stained for CD90 (93±2.6%, Fig. 5C and 5D) and CD105 (95±2.1%, Fig. 5E and 5F), which are the markers considered to be specific for the cells of mesenchymal origin. Furthermore, immunocyto-chemistry indicated that some cells were positively stained for Oct4 (12±3.1%; Fig. 6A and 6B) and Nanog (8±2.9%; Fig. 6C and 6D) but not for Sox2 (Fig. 6E and 6F) and Klf4 (Fig. 6G and 6H). To confirm the stromal cells of endometrium, our data clearly demonstrated that the endometrial cells are completely negative for any of the markers specific to epithelial cells such as CD9 (Fig. 5G and 5H) and EpCAM (Fig. 5I and 5J). In addition, the cells were stained with only secondary antibody (without primary antibody) serving as a negative control (Fig. 5K and 5L).

## DISCUSSION

In this study, it has been identified that CD146-positive cells as the markers of EnSCs are located in stroma of human endometrium in a perivascular location and near the glands. A perivascular location seems ideal for resident EnSCs to get signals from endothelial cells or blood stream in order to regenerate endometrium for each menstrual cycle. The present study also shows that CD146-positive cells reside in a more potential location in the basalis layer that could be a cell source of stem cells to avoid their loss during menstruation. Similarly, Schwab and Gargett^[^^16^^]^ have indicated that endometrial stromal stem cells are localized in perivascular cells of some vessels. Furthermore, it has been demonstrated that CD146 and STRO-1 are co-localized on the outer blood vessel wall in dental pulp^[^^22^^]^. 

Also, immunocytochemistry data confirmed that 28±2.3% of the cells express the CD146 marker in endometrial stromal cell culture at fourth passage. Therefore, we can conclude that the culture system in the current study could cause an increase in the percentage of the indicated marker during culture. Schwab *et al.*^[^^23^^]^ Identified that 11.0±3.0% of the isolated stromal cells at first passage expressed the CD146 marker. In comparison with our study, it seems that a long-term culture provides the strongest evidence for purification of human embryonic stem cells related to CD146-positive cells.

CD90 is one of the key mesenchymal markers used routinely in conjunction with CD105 to characterize characterize putative MSC populations. Similar to an earlier study, our finding indicated that CD90 and CD105 differentially stained the basalis and functionalis stroma^[^^23^^]^. It also strongly supports more potential location in the functionalis than basalis stroma. CD90 and CD105 were also identified in various tissues such as bone marrow and dental pulp^[^^24^^-^^27^^]^. 

**Table 1:** Immunohistochemical characteristics of stemness markers in human endometrial tissue sections

**Markers**	**Expression ** **in glandular epithelium**	**Expression** **in stroma**	**Basalis ** **stroma**	**Functionalis stroma**
CD146	-	++	++	+
CD90	-	+++	++	+++
CD105	-	+++	++	+++
Oct4	-	+	++	+
Nanog	-	+	++	+
Sox2	-	-	-	-
Klf4	-	-	-	-
CD9	++++	-	-	-
EpCAM	++++	-	-	-

The intensity of immunoreactive cells at different parts of endometrial tissue sections was divided into 5 grades from low to high (-, +, ++, +++, ++++).

**Table 2:** Proportion of endometrial stem cells markers in isolated cultured endometrial cells at fourth passage

**Markers**	**Endometrial** **stromal cells (%)**
CD146	28±2.3
CD90	93±2.6
CD105	95±2.1
Oct4	12±3.1
Nanog	8±2.9
Sox2	-
Klf4	-
CD9	-
EpCAM	-

Our immunocytochemistry data in this study demonstrated that CD90-positive cells were expressed highly (93±2.6%) in human endometrial stromal cells at fourth passage. However, Schwab *et al.*^[^^23^^]^ showed that 87.5±2.9% of the cell population in fresh endometrial cells were CD90 positive, which confirms more purification of MSCs during repeated cell passages.

Based on immunohistochemistry data, we found that low population of the cells expressed Oct4 and Nanog in endometrial tissue sections with higher Oct4 expression than Nanog, which correlates with our immunocytochemistry data. Although we made interesting observation that most of the Oct4 and Nanog-expressing cells could be seen in the basalis layer of endometrium, it may be concluded that human endometrial stromal stem cells reside in the basalis layer to avoid their loss and regenerate endometrium during menstruation. Similarly, previous reports showed Oct4 and Nanog proteins in the majority of endometrial samples^[^28^,^^29^^]^. Matthai *et al.*^[^^29^^]^ indicated the presence of Oct4-expressing stromal cells in normal human endometrium; however, they proposed that Oct4-expressing cells were also enhanced within the carcinoma tissue compared to the normal endometrial tissue. Thus, the increased number and the aberrant expression of pluripotency markers lead to abnormalities at various stages^[^^28^^,^^30^^]^. 

In the present investigation, CD9 and EpCAM were used as epithelial markers in human endometrial tissue and cultured endometrial cells. All the surface and glandular epithelial cells, but there were no stained stromal cells. The identification of these markers as negative markers in cultured endometrial cells at fourth passage confirms the importance of isolation of desired cell populations and purification of stromal cells. Therefore, our protocol could remove epithelial cells in order to purify stromal cells in the culture system.

The therapeutic potential of EnSCs is progressing; therefore, these cells are readily available sources of adult stem cells in the endometrium that could be a good alternative in reproductive biology, regenerative medicine, autologous stem cell therapy, and tissue engineering^[^^31^^,^^32^^]^.

In conclusion, this study supports a perivascular location for endometrial stromal stem cells and also provides that human endometrial stem cells and pluripotency markers may be localized more in basalis layer to regenerate endometrium during menstruation. Moreover, the immunostaining observations of endometrial cells at fourth passage are correlated with the immunohistochemistry data. 
